# Magnetic Nanoparticles for Rhodamine B Depletion in Wastewater—Theoretical and Experimental Approach

**DOI:** 10.3390/molecules30224447

**Published:** 2025-11-18

**Authors:** Gabriela Vochița, Andreea R. Fânaru-Balint, Anda Agavriloaei, Daniela Gherghel, Mihaela Răcuciu, Dorina Creangă

**Affiliations:** 1 Institute of Biological Research—Branch of National Institute of Research and Development for Biological Sciences, Lascăr Catargi Street, 47, 700107 Iași, Romania; gabriela.vochita@icbiasi.ro (G.V.); daniela.gherghel@icbiasi.ro (D.G.); 2 Physics Department, Alexandru Ioan Cuza University, Blvd. Carol I, Nr. 11, 700506 Iasi, Romania; fanaru.andreearoxana@yahoo.com (A.R.F.-B.); anda.agavriloaei@yahoo.com (A.A.); 3 Environmental Sciences and Physics Department, Faculty of Sciences, Lucian Blaga University of Sibiu, 5-7 Dr. I. Ratiu Street, 550012 Sibiu, Romania

**Keywords:** wastewater, magnetite nanoparticles, Rhodamine B, cytotoxicity, degradation products

## Abstract

We studied the impact of some magnetic nanoparticles on two wastewater models of Rhodamine B dye, with 5 µM and 10 µM concentrations. The magnetite nanoparticles, synthesized by the co-precipitation technique, having less than 20 nm diameter and typical crystallinity features, were used to treat the Rhodamine B solutions and the results were analyzed using spectral measurements. The biological efficacy of the photo-Fenton-like reactions underlying this wastewater treatment was assessed using the V79-4 fibroblast cell line of Chinese hamster. The MTT test (colorimetric method with 3-(4,5-dimethylthiazol-2-yl)-2,5-diphenyl tetrazolium bromide) was applied for the toxicity testing of Rhodamine B 10 µM and 5 µM, initially, and degraded with 8 g/L MNP and 10 mM hydrogen peroxide for 120 min of UV exposure, the cell viability decreasing to 57–59% and 69–74%, respectively, for the dose of 80 µL/mL. Morphological changes were identified by microscopy analysis, such as membrane disruption, cell content extravasation, apoptotic bodies, and also colored spherical inclusions suggesting non-metabolized dye solution aliquots. The simpler molecules consisting of Rhodamine B degradation products, i.e., benzoic acid, benzyloxyamine, and phthalic acid were analyzed for their theoretical reactivity through quantum chemical computational modeling, which revealed a significant chemical potential compared to Rhodamine B.

## 1. Introduction

Environmental pollution is a major global problem; thus, researchers are looking for solutions to counteract the factors that affect the soil, water, and air, which have a negative impact on the quality of life of people, biodiversity, and ecosystems. Drinking water is the most urgent and complex challenge facing humanity today, and is essential for life, health and economic development. An important aspect of this is the pollution of drinking water with chemical and toxic dyes. As a result of the processes specific to each industry, wastewater is generated—which, most often, contains dyes and is later poured into underground and above-ground water networks without any previously applied treatment.

Among the basic wastewater treatment procedures, adsorption technology continues to be a widely used method due to its versatility, low energy consumption, and relatively simple infrastructure and operational framework [1]. The main disadvantages of this method are that it transfers pollutants rather than destroying them, the saturation of adsorbents, and the potential for sludge production [2]. The increasing difficulty in addressing the complexity of industrial effluent composition has brought photocatalytic purification to the forefront as a rather promising approach offering the advantages of degrading refractory organic compounds that are resistant to conventional technologies [3,4]. However, there are some disadvantages related to high-energy light sources, issues related to practical application technologies, and operational costs to overcome barriers in large-scale water treatment applications [5].

In the search for improvements in advanced catalytic oxidation methods for wastewater purification, researchers have tested the introduction of superior heating methods with microwave irradiation, which can provide rapid and uniform heating without a temperature gradient. This can enhance the efficiency of the catalysis process and reduce energy consumption and reaction time, addressing various toxic components of industrial effluents that can be degraded more effectively with microwave-assisted catalysis, such as dyes, phenols, antibiotics, and pesticides [6]. Recent developments in photocatalytic materials, particularly nanocomposites, have demonstrated improved efficacy in wastewater treatment, underscoring the potential of this technology for effective pollutant removal [7].

To produce catalysts with highly efficient activity under ambient conditions [8], it is necessary to succeed to obtain well-controlled reaction parameters for the synthesis procedure.

Over time, from the need to find new methods to solve the previously mentioned problems of wastewater purification, scientists have come up with an innovative and effective solution, namely the use of magnetic nanoparticles (MNP).

Since the beginning of the study of magnetic nanoparticles, they have quickly found their role in the field of life sciences for their applications: in the targeted administration systems of some drugs; in the treatment of the most common disease nowadays, cancer, through induced hyperthermia; in medical and diagnostic imaging; and in gene therapy [9,10,11].

A wide variety of magnetic nanoparticles have attracted the interest of scientific, engineering, and industrial researchers. Some examples of magnetic nanoparticle use have been reported in the following domains: energy conversion, energy storage, batteries, and supercapacitors [12,13].

As wastewater treatment is a major global concern, considering its significant impact on human health and the environment; magnetic nanoparticles are being studied as a possible innovative and effective solution in this field due to their unique properties, such as their surface ratio—large volume, comparable in size to most of the analytes of interest, and the ease with which they are dispersed in solutions [14]. These properties make them extremely useful for removing contaminants from polluted water.

Although traditional methods of treating wastewater loaded with various toxic substances have been used for many years, as mentioned in [15]; it is imperative to develop newer, up-to-date techniques. We mention a couple of recent reports based on metal reactivity. Dye removal from wastewater was successfully applied using a mesoporous adsorbent yielded from pistachio shells enriched with iron [16], while an electrochemical method was applied to clear wastewater loaded with pesticides using metal oxide electrodes [17]. Promising results and shorter implementation times are also offered by nanotechnologies [18].

Recently, it was studied the use of a nanocomposite consisting of hydrogen titanate (H_2_Ti_3_O_7_) and maghemite, γ-Fe_2_O_3_, together with hydrogen peroxide (H_2_O_2_) for the adsorption of methylene blue molecules from aqueous solutions [19]. The performed spectral analyzes revealed favorable results regarding the success of nanotechnology applications. The methylene blue dye was also targeted by some researchers, who used microwave-treated silica nanoparticles obtained from rice husk/waste to remove the dye from aqueous media [20]. In [21], the authors presented the use of the photo-Fenton technique, with Fe_2_O_3_/Au/SiO_2_ as a catalyst, as a treatment in the cleaning of waters polluted with methyl orange dye [21]. Another toxic dye that has been the basis of various studies in the literature is Sudan dye, whose adsorption from contaminated water using magnetite nanoparticles, either alone or associated with aminosilane [22].

Rhodamine B is a synthetic dye, significantly exploited, especially in the textile industry, but not only, due to its fluorescent characteristics even at very low concentrations. More recently, it is a particularly promising fluorescent marker in the continuously expanding biotechnologies. The large-scale presence of Rhodamine B molecules in the environment of water reservoirs, both surface and underground, can have dangerous effects on human and animal health. Rhodamine B is known to cause toxicity and teratogenicity in aquatic species, such as zebrafish [23]. As early as 1967, the question of the toxicity of the Rhodamine B dye was raised, through the study presented in [24], joining more recent studies [25,26].

Various methods have been sought over time to decontaminate industrial effluents from Rhodamine B dye. Carbon-based materials have been studied in this process [27], demonstrating the effectiveness of kaolin and organic bentonite composites. The photocatalytic degradation of Rhodamine B using nanocomposites was reported in [28]. As for the other dyes, already mentioned, the applications of nanotechnologies are useful for the extraction of Rhodamine B molecules from contaminated water. In the literature, we found a study [29] in which they presented the action of SiO_2_/Ag nanoparticles used as a catalyst in the sonocatalytic degradation of Rhodamine B from aqueous solution. Other authors studied the discoloration of aqueous solutions of Rhodamine B under the influence of copper ferrite nanoparticles (CuFe_2_O_4_) and hydrogen peroxide [30]. The efficacy of activated carbon prepared from palm residue from agricultural biomass and coated with magnetic nanoparticles (Fe_3_O_4_) was investigated in [31]. Furthermore, satisfactory results in their study on the degradation of Rhodamine B with the help of magnetite nanoparticles, Fe_3_O_4_, coated with humic acid were presented in [32]. All these studies, but not only these studies, have increased the interest of researchers in looking for techniques, as modern and efficient as possible, to incorporate nanotechnology in their practice that focuses on the decontamination of polluted waters. As for Rhodamine B pollution, there is concern that the impact of this dye on environmental waters can affect many living beings, including humans, so that, as early as 1987, Rhodamine B was included on the list of potential carcinogens by the International Agency for Research on Cancer [33]. Experimental studies on in vitro cell cultures have shown that Rhodamine B at concentrations of the order of tens of micrograms per milliliter induces the slowing of proliferation after 48 h of incubation [34]. It was found that some cells were affected, showing irregular shapes, with nuclei modified in the sense of their degeneration.

The treatment of water loaded with Rhodamine B was found to reduce the level of dye concentration by removing or decomposing it into simpler products [35], up to complete mineralization (water and carbon dioxide), among conventional chemical methods also considering magnetic nanoparticle use.

We present the influence of magnetite nanopowders on the effects of Rhodamine B solutions on fibroblast cell cultures analyzed from quantitative and qualitative viewpoints.

We studied the toxicity of Rhodamine B and magnetic-nanoparticles-degraded Rhodamine B in V79-4 cells, which we did not find to be investigated in the available literature. We described the theoretical reactivity and chemical strength of the Rhodamine B degradation products, which still seem to represent cytotoxic agents. Since these products were identified [35] after treatment of 0.1 mM Rhodamine B with 8 mM hydrogen peroxide and 8 mM zero-valent iron, we chose to work with the same order of magnitude concentrations of dye and hydrogen peroxide but using magnetite, which is more available than zero-valent iron, and adding ultraviolet exposure. The benefits of the proposed method are given by the availability, low toxicity, and low cost of magnetic nanoparticle synthesis and mainly by the relatively low efficiency of other treatment procedures, such as those based on biological processes [36] or classical coagulation/flocculation.

The novelty of our study lies in the evaluation of the photodegradation of Rhodamine B using magnetic nanoparticles and by correlating the results of the photo-Fenton reaction efficiency with those of quantum chemical modeling and biological assays. Compared to previously published research that specifically analyzes bleaching efficiency, our methodology established a direct correlation between the physicochemical degradation profile and cytotoxic effects. This approach also takes into account the fact that certain degradation products may retain some reactivity and biological significance for degraded dye solutions. We focused on demonstrating the effects of photo-Fenton degradation through the results of cytotoxicity tests of the studied dye before and after treatment with nanoparticles, which we propose as a new experimental demonstration of the utility of nanoparticles in ameliorating wastewater toxicity.

## 2. Results and Discussion

### 2.1. Magnetic Nanoparticles Characterization

We considered that, usually, the ability of nano-magnetite to catalyze the degradation of pollutants by advanced oxidation processes, such as photo-Fenton reactions, was demonstrated by first characterizing magnetite nanoparticles by techniques such as XRD and TEM microscopy, and then demonstrating the degradation of contaminants by measuring the percentage of pollutant removal.

**Figure 1:**
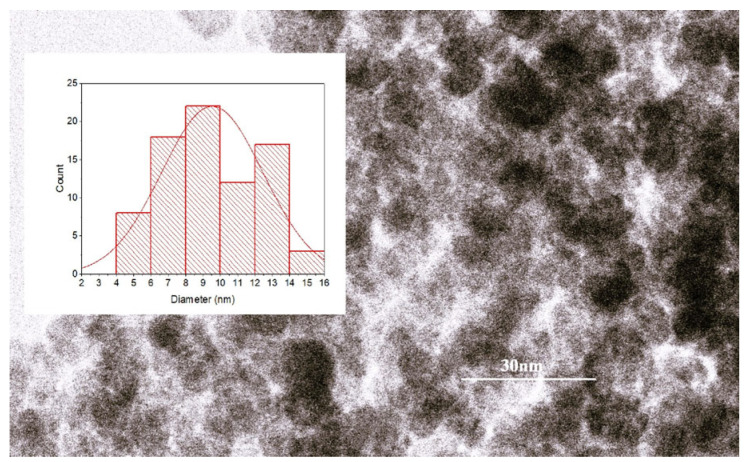
The results of TEM analysis with size histogram fitted with normal statistical function (red line—the statistical fitting curve).

According to the TEM investigation, the particles from the sample studied in this work had symmetrical quasi-spherical shapes and a mean size of 10.8 nm, as resulted from the analysis of the size histogram (Figure 1), thus providing a high total surface area for interaction with the dye molecules.

**Figure 2:**
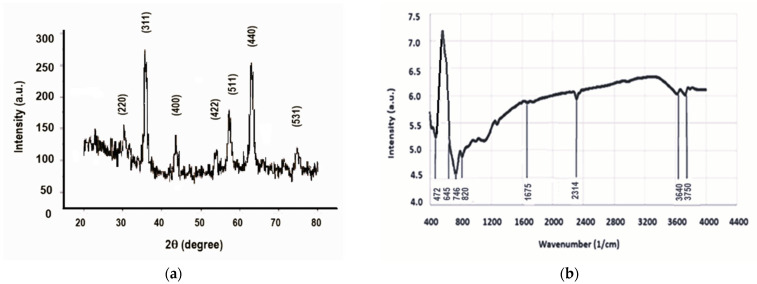
(**a**) The results of XRD investigation; (**b**) the vibration spectrum.

The recorded diffractogram (Figure 2a) shows the diffraction maxima characteristic of the magnetite crystallization planes (220), (311), (400), (511), (440), and (533) (JCPDS:19-0629) [37], without any other impurities. The highest diffraction peak (for the crystallization plane (311)) gave the crystallite size of 10 nm, which is in agreement with the literature [38].

The FTIR analysis (Figure 2b) showed vibrations corresponding to iron oxide spinel crystals between the wave numbers 400 cm^−1^ and 900 cm^−1^, more precisely, the band characteristic of the vibrations of the (Fe-O) bond at 472 cm^−1^. The relatively low-intensity maximum at 1675 cm^−1^ could be attributed to the bending deformation vibrations of the OH from certain traces of water adsorbed on the nanoparticles [39]. During the synthesis, the presence of carbon dioxide, which binds to iron oxides, gave a low-intensity vibrational maximum at 2314 cm^−1^ [40]. Similarly to the literature results, the stretching vibrations of OH from water traces were found at approximately 3640 cm^−1^ and 3750 cm^−1^, respectively [41].

These results prove that the magnetite synthesized by us is close to the expected standard ones. The catalytic action of magnetite nanoparticles with small size and typical crystallinity features for the wastewater treatment over a wide pH range has been reported by many authors, who focused on efficient Fenton catalysts for the generation of •OH for the degradation of organic pollutants [42].

### 2.2. Rhodamine B Solutions Photodegradation

Photo-Fenton reactions are advanced oxidation processes that involve a combination of a catalyst (usually an iron-based material), hydrogen peroxide, and UV or visible light, resulting in the production of highly reactive hydroxyl (•OH) radicals and other oxidizing species for the decomposition of pollutants. Using incident light or UV radiation, the reactions of hydrogen peroxide with an iron catalyst, which generates highly reactive hydroxyl radicals, are more efficient [43,44].

In our experiment, iron ions on the surface of magnetite nanoparticles acted as catalysts for the decomposition of hydrogen peroxide. The reactive •OH radicals generated in this way were supplemented by those resulting from water photolysis under UV-C exposure in advanced oxidation processes to degrade Rhodamine B, as resulted from our measurements.

**Figure 3:**
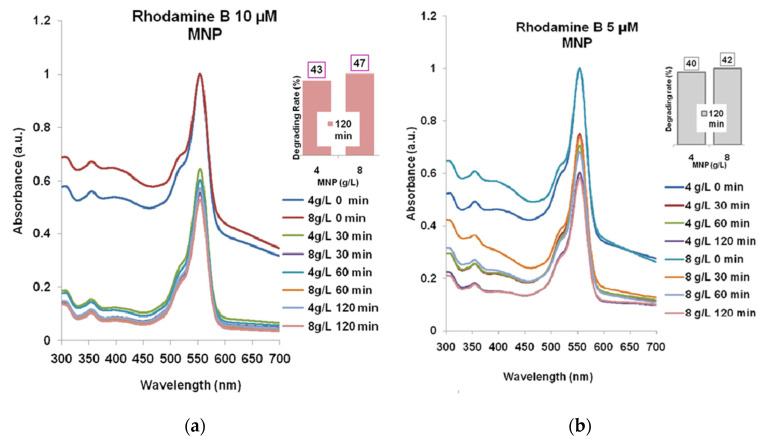
The Rhodamine B degrading with MNP for 30–60–120 min: (**a**) Rhodamine B 10 µM; (**b**) Rhodamine B 5 µM. The absorption spectra for 4 g/L and for 8 g/L MNP, respectively, are normalized relatively to the maximum absorption (at 554 nm) for the spectra recorded at t = 0 min.

The effect of magnetite nanoparticles (MNP) on the two wastewater model solutions is presented in Figure 3a,b.

One can see that the solutions stirring for 30–60–120 min resulted in the decreasing of absorbance at 554 nm. In the case of Rhodamine B 10 µM (Figure 3a), the lowest absorbance was recorded for 8 g/L MNP like in the case of Rhodamine B 5 µM (Figure 3b), after 120 min which means a degradation efficacy of 47% and 42%, respectively.

The pH of the studied solutions was estimated with indicator paper; in the case of wastewater initial solutions with Rhodamine B, the result was neutral (7–7.5) due to the very low concentrations of the dye, 5 µM and 10 µM, respectively, and because Rhodamine B is a weak acid/base. At micromolar concentrations, the contribution of Rhodamine B to the overall pH of the solution seems to have been negligible.

The addition to the initial solutions of 10 or 20 mM hydrogen peroxide, which itself is not a strong acid or base and decomposes in water, has slightly shifted the pH to acidic values (6.0–6.5 without a clear correlation with the stirring time) and no correction was applied to the dye solutions.

**Figure 4:**
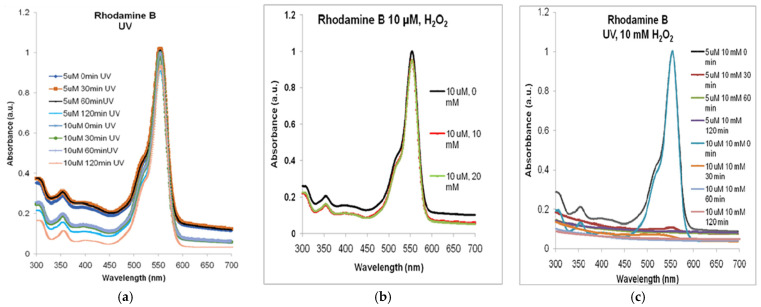
The absorbance spectra of Rhodamine B in the lack of MNP: (**a**) only exposure to ultraviolet radiation of 5 µM and 10 µM Rhodamine B (spectra normalized to the maximum absorption (at 554 nm, at t = 0)); (**b**) after 120 min of stirring with H_2_O_2_ only (spectra normalized to the maximum absorbance for 0 mM); (**c**) after combined treatment with H_2_O_2_ and UV. Normalized spectra.

The exposure of the two solutions to ultraviolet radiation (Figure 4a), in the lack of MNP or H_2_O_2_, also resulted in a less than 10% decrease in the absorption at 554 nm: 9% for Rhodamine B 5 µM and 7.5% for Rhodamine B 10 µM. Although at smaller wavelengths, the absorbance decreased significantly more in both dye solutions after 120 min, it seems that hydroxyl free radicals were produced in rather low concentrations and their degrading action needs to be sustained by MNP and hydrogen peroxide.

As exemplified in Figure 4b, for Rhodamine B 10 µM, less than 5% decrease could be observed in the absorption spectra for either 10 or 20 mM of H_2_O_2_ compared to non-treated solution (similar situation being found for Rhodamine B 5 µM).

The decomposition of Rhodamine B was practically not observable after treatment with only hydrogen peroxide in, as similarly shown in [45], where the Rhodamine B concentration and that of hydrogen peroxide were of a similar order of magnitude as in our case. The authors of [35] found that for H_2_O_2_ concentrations lower than 10 mM, the degradation efficiency of 100 µM Rhodamine B is higher than for H_2_O_2_ concentrations above 10 mM (as we used), and the degradation percentage probably decreased because H_2_O_2_ becomes an •OH scavenger and forms •HO_2_ with a lower oxidation capacity than •OH.

In photodegraded dye solutions, we did not notice consistent changes for pH var-ying in the range of 5.0–6.5. Without a catalyst, the effect on pH is often minimal while in the presence of the catalyst, the reaction complexity increases the degradation efficacy [46]. Other authors have shown that different pH levels (3–9) of the initial Rhodamine solution gave quite similar heterogeneous Fenton oxidation effects, with maximum decolorization at pH 5.4 [47]. This seems to be a considerable benefit, as pH adjustment became unnecessary and promotes practical applications.

In Figure 4c, we present the synergic effects of the two sources of •OH free radicals, the hydrogen peroxide and the UV radiation, that, together—but in the lack of MNP, in the case of our experimental conditions—decomposed, almost totally, the Rhodamine B solution within less than 30 min.

The results were practically the same for the two dye solutions studied by us. Although this process is an effective advanced oxidation reaction path for degrading Rhodamine B, we further studied the catalytic effect of magnetic nanoparticles in combination with hydrogen peroxide and UV radiation.

**Figure 5:**
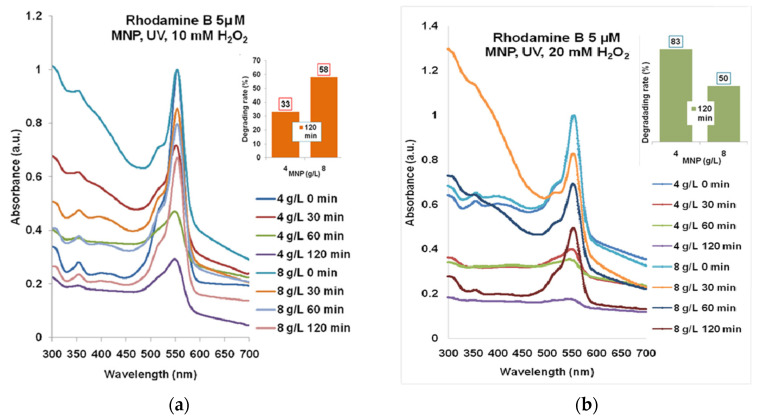
The results of combined treatment of Rhodamine B 5 µM with MNP, UV, and hydrogen peroxide: (**a**) 10 mM; (**b**) 20 mM. Normalized spectra.

According to the spectra presented in Figure 5a, the maximum degrading effect of 58% was obtained for Rhodamine B 5µM treated with 4 g/L MNP and 10 mM H_2_O_2_ for 120 min UV exposure. In Figure 5b, one can see the best combination of 8 g/L MNP and 20 mM H_2_O_2_ and 120 min of UV exposure which gave over 83% degrading rate of Rhodamine B 5 µM (maximum absorbance decreasing to approximately 0.05).

**Figure 6:**
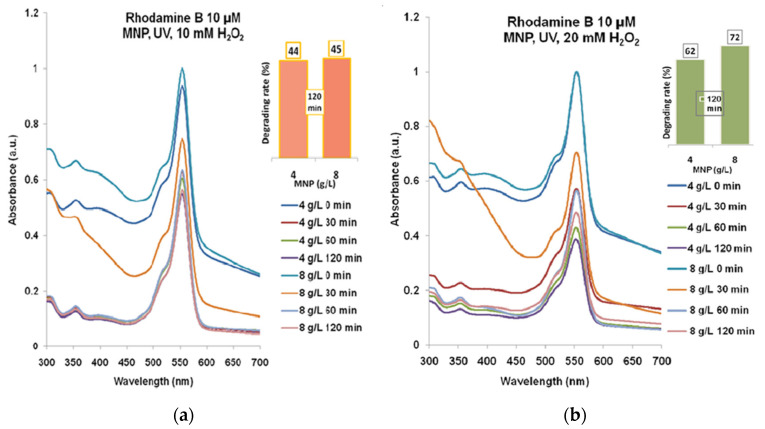
The results of combined treatment of Rhodamine B 10 µM with MNP, UV, and hydrogen peroxide: (**a**) 10 mM; (**b**) 20 mM. Normalized spectra.

The highest degrading rate for Rhodamine B 10 µM (Figure 6a) that has resulted from the combination of 4 g/L MNP and 20 mM H_2_O_2_ after 120 min of UV exposure was almost 44%. According to Figure 6b, the degradation with 20 mM H_2_O_2_ led to 62–72% efficacy for 4 g/L and 8 g/L MNP for 120 min UV exposure.

These results can be related to the degradation of the studied molecule through the attack of •OH radicals released due to the catalytic effect of iron ions at the surface of the magnetic nanoparticles. Some authors have demonstrated the generation of free •OH radicals with ESR investigation of dye solution photodegraded with magnetite nanoparticles and hydrogen peroxide [48], evidencing the catalytic action of magnetite nanoparticles.

**Figure 7:**
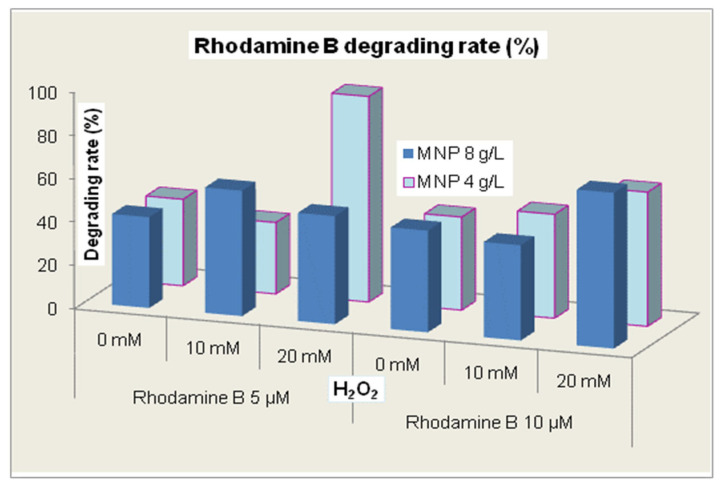
The rate of Rhodamine B degradation during 120 min of treatment.

In Figure 7, we present a summary of all the results obtained during the experimental study. Generally, there was no direct correlation between the dye concentration and the other reagent concentrations, suggesting the complexity of the photodegradation mechanisms. For Rhodamine B 10 µM, the effects of the two MNP concentrations were rather similar for each concentration of hydrogen peroxide while for Rhodamine B 5 µM, a distinct effect was evidenced for the combination of 4 g/L MNP and 20 mM hydrogen peroxide, which seems to shape an optimum set of reaction conditions.

**Figure 8:**
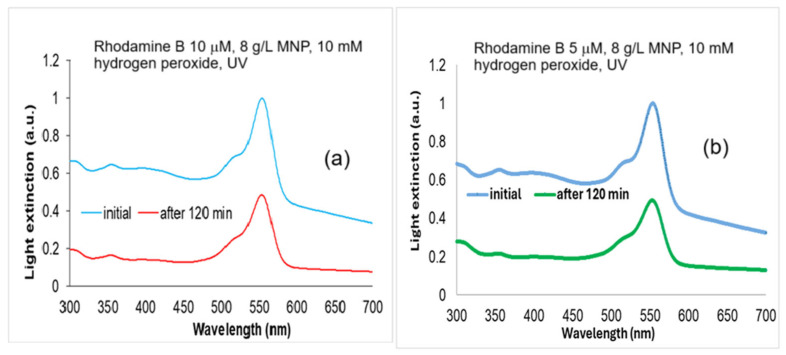
The absorption spectra of Rhodamine B solutions: (**a**) Rhodamine B 10 µM initial and degraded with 10 mM hydrogen peroxide, and 8 g/L MNP for 120 min UV exposure; and (**b**) Rhodamine B 5 µM initial and degraded with 10 mM hydrogen peroxide, and 8 g/L MNP for 120 min UV exposure. Normalized to maximum absorbance at 554 nm in the spectra of non-degraded samples.

For the biological tests, we considered the Rhodamine B 5 µM and 10 µM solutions (Figure 8) treated with 10 mM H_2_O_2_ and 8 g/L MNP for 120 min UV exposure, with degradation rates of 58 and 44%, according to Figure 5b and Figure 6a.

For large-scale treatment, the limitation of the proposed technique concerns the control of homogeneous mixing of Rhodamine B with the magnetic nanoparticles. When maximum intimate contact of reagents is fulfilled, then it is expected that large-scale process could be conducted successfully. Also, the UV-C irradiation of large vessels containing large solution volumes could raise practical issues.

Aiming to provide not only quantitative but also qualitative results related to the degradation products that were described by computational modeling, we worked with similar concentrations of Rhodamine B and hydrogen peroxide like in [35], where the authors identified, experimentally, the main final degradation products of degraded Rhodamine B. We replaced zero-valent iron with the much more available magnetite, in increased concentration, and we added ultraviolet radiation exposure and 10 mM H_2_O_2_, and in such conditions the degradation rates were between 33% and 58% after 120 min of exposure to UV-C.

### 2.3. Quantum Chemical Parameters of Rhodamine B and Its Degradation Products

**Figure 9:**
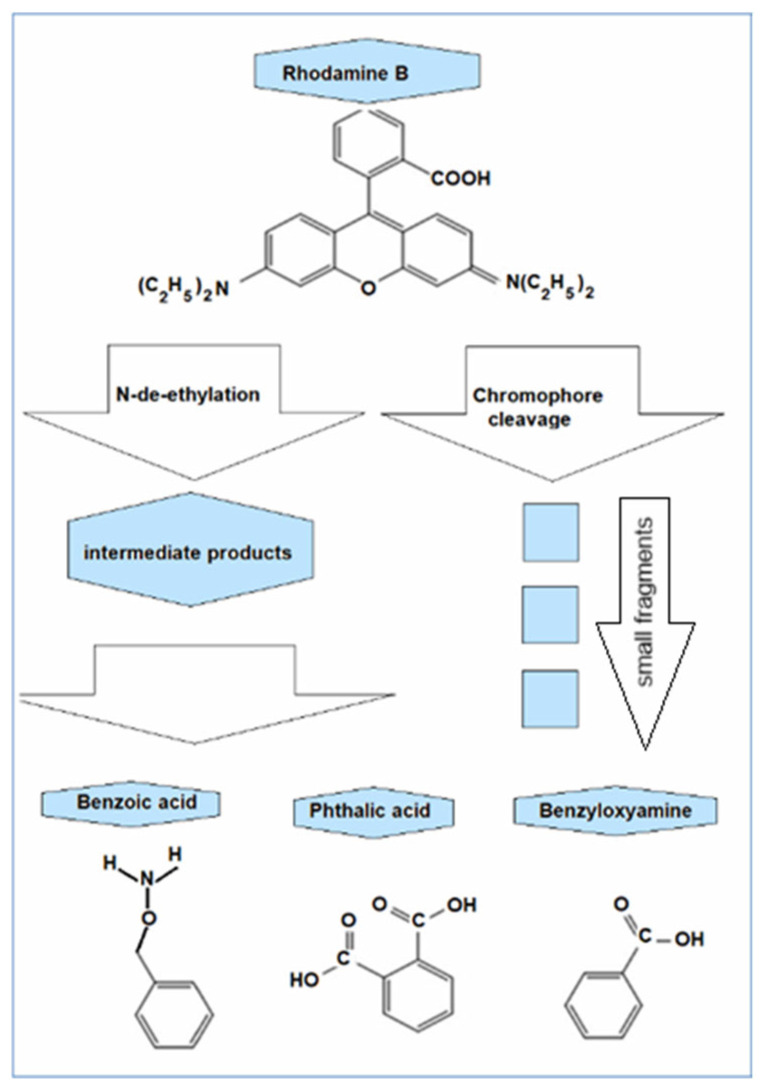
The degradation products of Rhodamine B (C_28_H_31_ClN_2_O_3_) identified by chromatography: benzoic acid (C_7_H_6_O_2_), benzyloxyamine (benzylhydroxylamine, C_7_H_9_NO), and phthalic acid (C_8_H_6_O_4_) modified after [35].

The studies reported in [35] focused on the degradation of Rhodamine B by hydroxyl radicals released though Fenton processes and propose two ways of modification: by de-ethylation and by breaking the chromophore, with the mention that the breaking of the chromophore is the main way of fragmentation of Rhodamine B into simpler compounds, each with a single aromatic ring: the benzoic acid, the benzyloxyamine, and the phthalic acid (Figure 9).

Further decomposition could simply lead to methane, ammonia, carbon dioxide, and water.

One could describe the Rhodamine B photodegradation through the next processes, triggered by •OH radicals released from water photolysis and the decomposition of unstable hydrogen peroxide.

The most direct decolorization pathway of Rhodamine B molecule is its decomposition through the •OH attack at the central carbon of the chromophore (the conjugated oxygen hetero-anthracene). By splitting the molecule at the level of chromophore, uncolored benzene-ring compounds like benzoic acid (C_7_H_6_O_2_), phthalic acid (C_8_H_6_O_4_), and benzyloxyamine (C_7_H_9_NO) are mainly produced that further suffer •OH disruptive attack.

•OH + C_28_H_31_ClN_2_O_3_ → C_7_H_6_O_2_ + C_7_H_9_NO + C_8_H_6_O_4_ + smaller fragments.

•OH + C_7_H_6_O_2_ + C_7_H_9_NO + C_8_H_6_O_4_ → CO_2_, H_2_O, NH_4_^+^, etc.

They were identified using GC-MS analysis in [35]. Or, the action of •OH radicals could first be directed at the ethyl groups that are separated from the dye molecule, transforming it into Rhodamine 110 (C_20_H_15_ClN_2_O_3_), still-colored but with the absorption maximum shifted to blue [49], which is further attacked by •OH radicals, subsequently resulting in the same benzene-ring compounds.

•OH + C_28_H_31_ClN_2_O_3_ → C_20_H_15_ClN_2_O_3_ → C_7_H_6_O_2_ + C_7_H_9_NO + C_8_H_6_O_4_ + smaller fragments.

For longer time duration (after 120 min), most of the intermediate benzene-ring products resulted from either of the reaction pathways are gradually degraded into smaller compounds that are eventually mineralized into CO_2_, H_2_O, NH_4_^+^, etc.

When the Rhodamine B solution is partially photodegraded, the remaining coloration is rather due to undegraded molecules coexisting with the three smaller transparent compounds [50] than to its still-colored intermediates.

**Figure 10:**
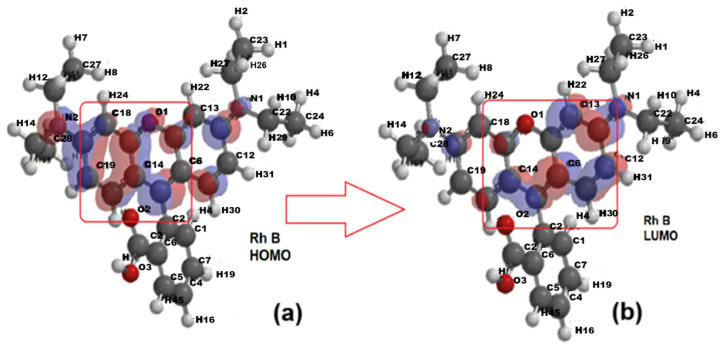
The electron density for the optimized structure of Rhodamine B (Rh B): (**a**) the ground state—HOMO (Highest Occupied Molecular Orbital); (**b**) the excited state—LUMO (Lowest Unoccupied Molecular Orbital.

The optimized structures were generated and the electric dipole moment values were calculated for Rhodamine B (10.3 D) in concordance with [51] and benzoic acid (2.25 D), benzyloxyamine (0.25 D), and phthalic acid (3.13 D). The density distributions were then plotted for the ground (HOMO) and excited (LUMO) states as well as the ionization potential (IP) and electrostatic potential (EP) maps. Figure 10a,b show the electron density distribution for the ground state (HOMO—Highest Occupied Molecular Orbital) and the excited state (LUMO—Lowest Unoccupied Molecular Orbital) of Rhodamine B. This molecule is a rather symmetrical structure with three aromatic rings, the central one containing the O1 atom and being bound to a fourth ring, carboxyl substituted.

**Figure 11:**
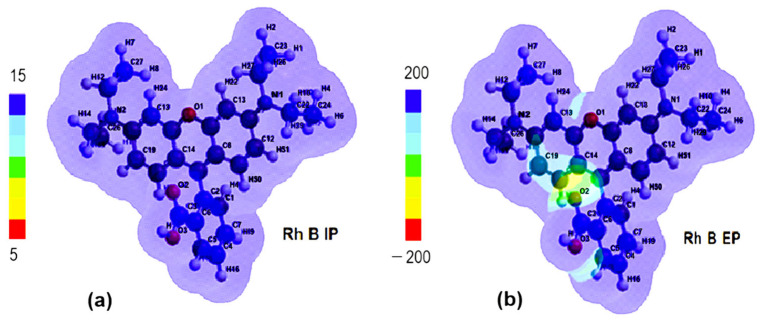
(**a**) Ionizing potential (IP) map and (**b**) electrostatic potential (EP) map of Rhodamine B (Rh B).

With the transition from the ground state to the excited state, the electronic density shifts from the aromatic ring on the left toward the right-side ring containing the O1 atom. The comparison of the distribution maps of IP and EP (Figure 11a,b) shows that IP is positive and uniformly distributed (blue coloration) but EA shows not only blue coloration, corresponding to the positive region where the nucleophilic attack could occur, but also a greenish yellow region representing the site with negative EA values, where electrophilic attack is more likely.

**Figure 12:**
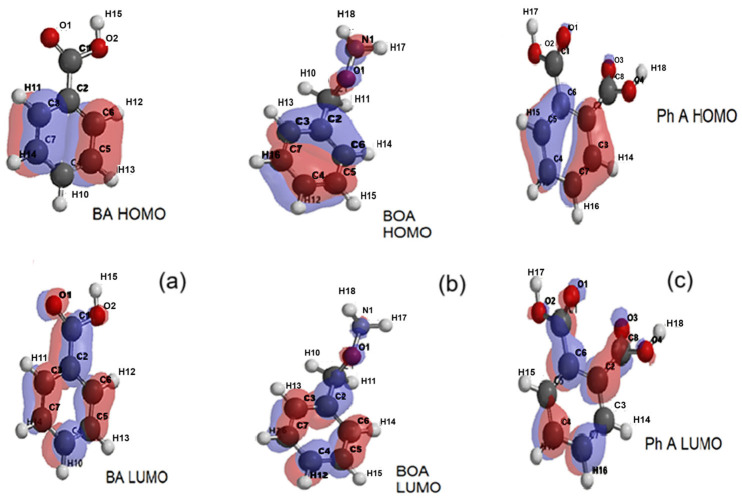
The representations of electron density distributions for the frontier orbitals of (**a**) benzoic acid (BA), (**b**) benzyloxyamine (BOA), and (**c**) phthalic acid (Ph A).

From Figure 12a, it can be seen that, for benzoic acid, in the ground state (HOMO), the electron density is concentrated more on the aromatic ring while, for the excited state, (LUMO), a redistribution occurs towards the carboxyl substituent (COOH). According to Figure 12b, in the ground state (HOMO), the spatial distribution of the electron density of benzyloxyamine includes both the aromatic ring and the amide substituent, CONH_2_, while in the excited state (LUMO), this distribution is mostly concentrated on the outline of the aromatic ring. The redistribution of the electron cloud density of phthalic acid between the ground state (Figure 12c) and the excited one occurs in reverse, from the aromatic ring to the carboxylic substituents.

**Figure 13:**
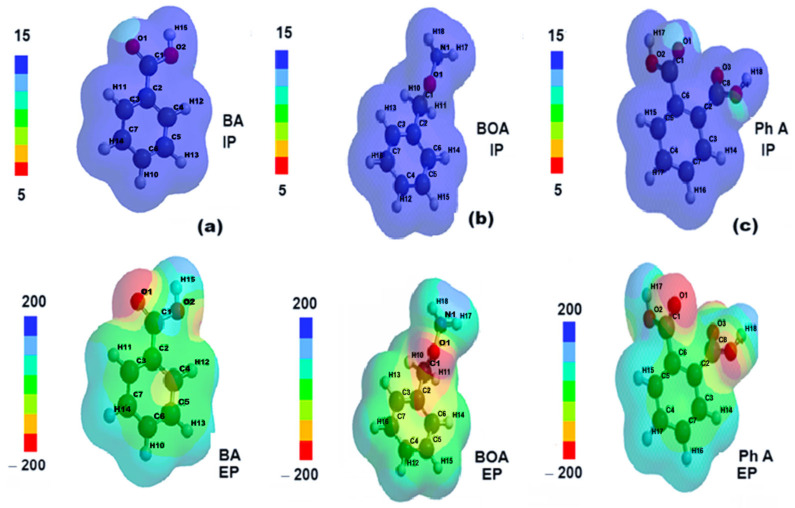
The distributions of ionizing potential and electrostatic potential of (**a**) benzoic acid (BA), (**b**) benzyloxyamine (BOA), and (**c**) phthalic acid (Ph A).

According to Figure 13a–c, the distributions of the ionization potential on the surface of the molecules of the degradation products are quite uniform, with positive values corresponding to the relation E_HOMO_ = −IP (−10.13 eV for benzoic acid, −9.66 eV for benzyloxyamine, and −10, 46 eV for phthalic acid).

The electrostatic potential maps show the molecular sites of low electronegativity—the carboxyl (COOH) oxygen in the BA structure (Figure 13a), the amide oxygen in the BOA structure (Figure 13b), and the oxygens in the two carboxyl substituents of Ph A (Figure 13c) as reddish areas where electrophilic attack is more likely.

The energy gap between the HOMO and LUMO frontier orbitals of a molecule is related to its reactivity and photodegradation efficiency.

**Figure 14:**
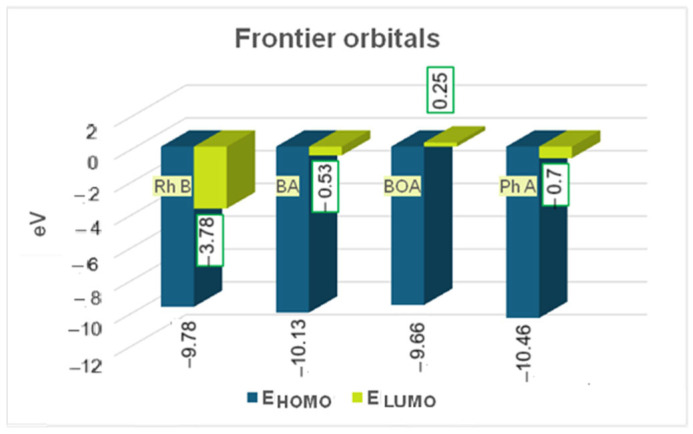
Energy of frontier orbitals of Rhodamine B (Rh B) and of its degradation products (BA- benzoic acid, BOA-benzyloxyamine, and Ph A-phthalic acid).

A higher HOMO–LUMO energy gap generally leads to lower efficiency of molecule photodegradation because it requires more energy to excite an electron from the highest occupied molecular orbital to the lowest unoccupied molecular orbital [52]. Generally, an HOMO–LUMO energy gap is considered low if its value is under 5 eV, and a gap below 0.5 eV can be considered exceptionally low [53]. The smaller the gap, the less stable the compound and more reactive. We have computed about 6 eV for Rhodamine B and 9.6 eV for BA (benzoic acid), 9.9 eV for BOA (benzyloxyamine), respectively, and 10.4 eV for Ph A (phthalic acid) which means Rhodamine B is rather reactive and photodegradable compared to its rather stable degradation products (Figure 14). The energy gap (E_HOMO_ − E_LUMO_) of Rhodamine B is significantly higher than for all three degradation products since their E_LUMO_ values are significantly lower than for Rhodamine B although the E_HOMO_ values are only slightly different (Figure 14).

The chemical reactivity descriptors such as chemical hardness (*η*) and chemical potential (*µ*)—in terms of internal energy and in terms of electrostatic potential, i.e., global electrophilicity (*ω*) and absolute electronegativity (*χ*), were calculated with the energies of the frontier orbitals, HOMO and LUMO:

(1) $$\eta \left(eV\right)=\left(1/2\right)\left({-E}_{HOMO}+E_{LUMO}\right)$$

(2) $$µ(eV)=(1/2)\;(E_{HOMO}+E_{LUMO}\;)$$

(3) $$\chi (eV)=(1/2)\;(-E_{HOMO}-E_{LUMO}\;)$$

(4) $$\omega (eV)=\left(\frac{{µ}^{2}}{2\eta }\right)$$

**Figure 15:**
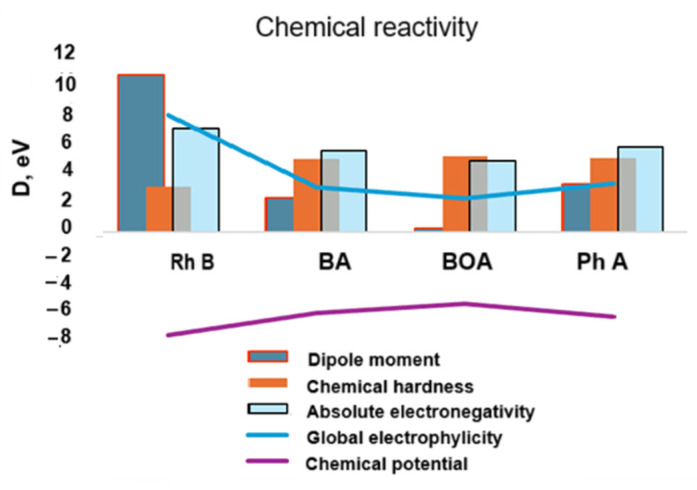
Theoretical chemical activity of the studied structures in terms of internal energy (chemical potential vs. chemical hardness) and of electrostatic type energy (global electrophylicity vs. absolute electronegativity).

The global electrophilicity (Figure 15) decreases significantly for the degradation products compared to Rhodamine B which confirms the increase in their chemical hardness, i.e., chemical stability. The absolute electronegativity and, simultaneously, the chemical potential also have lower absolute values in the degradation products compared to Rhodamine B.

The higher value of the chemical hardness indicates that the chemical stability increases and, thus, the degradation structures become less reactive and, as one could hypothesize, they exhibit stable toxicity [54].

It can be concluded that the molecular stability, in terms of internal energy (chemical potential and chemical strength) as well as electrostatic type energy (electrophilicity) is amplified for the analyzed degradation products: benzoic acid, benzyloxyamine, and phthalic acid. In the next section, we present the results of some cytotoxicity tests of Rhodamine B and its degraded solutions carried out on mammalian cell cultures.

Given that a smaller HOMO–LUMO energy gap is generally associated with higher chemical reactivity and bioactivity, the molecule is more easily excited and can participate in more chemical reactions, which can lead to increased cytotoxicity, while on the other hand, in the case of Rhodamine B, the frontier orbital energy gap is relatively large (6 eV), hence its cytotoxicity can be considered moderate at least at micromolar concentrations.

### 2.4. Cytotoxicity Test Results

**Figure 16:**
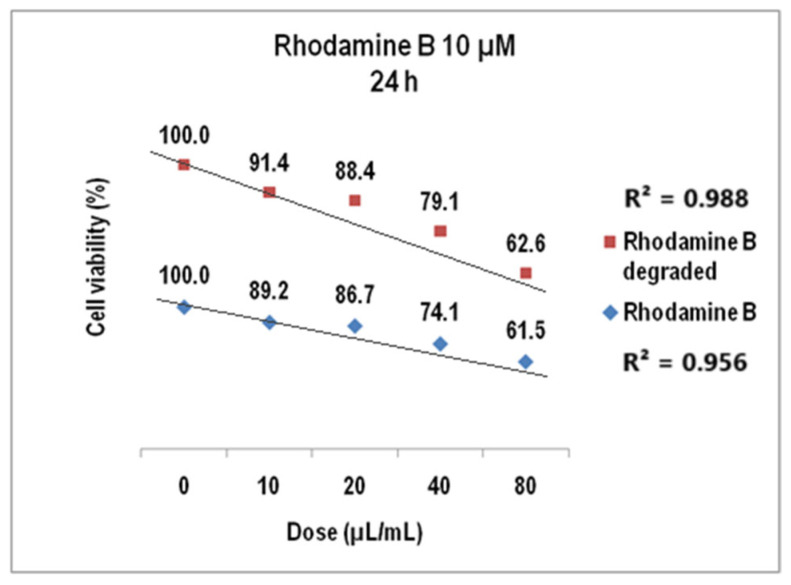
The viability of V79-4 cells at 24 h for Rhodamine B 10 µM before and after degradation.

In Figure 16, the V79-4 cell viability is presented for 10 µM Rhodamine B and Rhodamine B degraded at 24 h. For doses of 10–80 µL/mL, the viability at 24 h decreased to about 61% (average StDev of 2.5%) and to over 62% for the degraded dye (average StDev of 2.1%).

We evidenced that cell viability values decrease linearly to the increase in the volumetric dose with linear correlation coefficients of 0.95 for undegraded Rhodamine B 10 µM and 0.98 for the corresponding degraded dye solution.

**Figure 17:**
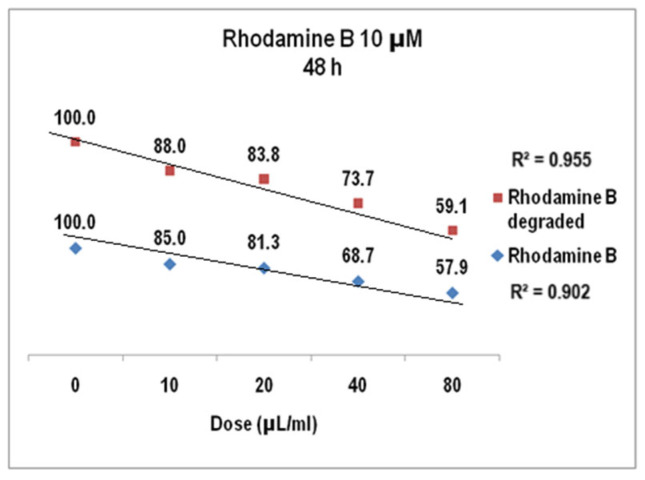
The response of V79-4 cells at 48 h to the supply with Rhodamine B 10 µM before and after degradation.

The cell viability assayed in the same biological samples at 48 h (Figure 17) showed a decrease of up to 57.9 for Rhodamine B 10 µM (StDev 1.6%) and the dose of 80 µL/mL while the cytotoxic effect of the degraded Rhodamine B 10 µM was given by cell viability of 59% for the same dye solution (1.5 StDev %), with the linear correlation coefficients also over 0.9 (0.902 for Rhodamine B 10 µM before degradation and 0.96 after degradation).

**Figure 18:**
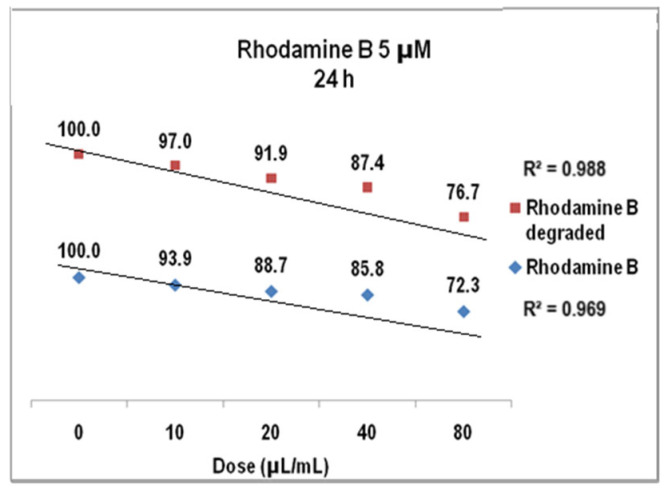
The graph of V79-4 cells viability at 24 h for the Rhodamine B 5 µM before and after degradation.

**Figure 19:**
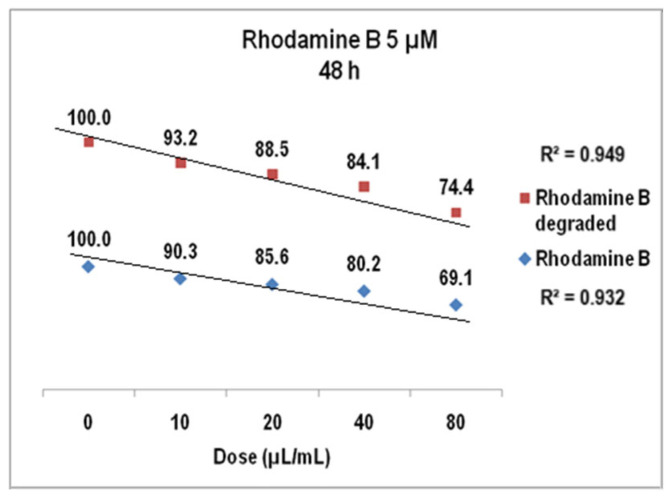
The graph of V79-4 cells viability at 48 h for the Rhodamine B 5 µM before and after degradation.

The results of cell viability assay for Rhodamine B 5 µM at 24 h presented in Figure 18 were also linear dose–effect curves and were still found with higher correlation, both R^2^ values being over 0.95 (0.96 for Rhodamine B before degradation and 0.98 after degradation). The difference between the cell viability at the highest dye dose is higher than in previous situation, i.e., 72% versus 76%. Standard deviations were of 2.32% (undegraded Rhodamine B 5 µM) and1.76% (degraded Rhodamine B 5 µM), respectively. For Rhodamine B 5 µM (Figure 19), cell viability at 48 h decreases to approx. 74% for the degraded solution (average StDev of 1.4%) and to 69% for the undegraded one (average StDev of 2.3%), respectively, 5 µM. The linear trend is maintained, with R^2^ = 0.93 and R^2^ = 0.94, respectively, and the significant statistic difference between the two arrays of values was evidenced with *p* ˂ 0.001 according to ANOVA test.

The solutions of degraded Rhodamine B would be expected to show significantly lower toxicity as a result of the decrease in dye concentration. However, the results show that cell viability is only slightly higher for both 10 µM degraded Rhodamine B and 5 µM degraded Rhodamine B compared to the original, non-degraded solutions.

To give an interpretation of this fact, we assumed that the final products formed by de-ethylation and/or cleavage of the chromophore under the action of OH radicals were still present in the solutions with degraded Rhodamine B. These end products no longer contribute to the coloration of the solution as they no longer have the Rhodamine B chromophore. Due to their lower reactivity (higher chemical strength) compared to Rhodamine B, it is expected that they contribute less to the impact of the degraded solutions on the tested cell cultures.

The 10 mL vials with degraded dye were held on powerful laboratory magnets until the entire volume remained completely transparent upon visual inspection, and only then did we very carefully extract aliquots of degraded solution (10 to 80 µL for 1 mL culture medium) from the top layer of the solution vial—but we cannot state that absolutely no traces of nanoparticles were present in the subsequently analyzed solution. However, the main effect is that of the Rhodamine B molecules and its degrading products, which dominate the sample content.

Experiments by other authors, who discussed the influence of magnetite on cell viability, showed that a small fraction of the initial powder concentration could have some inhibitory effects on different cell types (Table 1).

Indeed, for magnetic nanoparticle concentrations ten times lower than that used in our study, toxic effect was reported in HeLa cancer cells [55], while in [56], it was revealed the cytotoxic influence on the neural PC12 cells. Other authors have evidenced the cytotoxicity on Caco-2 and MCF-7 human cancer cells [57] or have demonstrated that magnetite nanoparticles induced the diminution of cellular viability [58], at concentrations of about a thousandth of the initial concentration of magnetic nanoparticles used by us, which could possibly remain as traces in the final dye solution, and could contribute significantly to decreasing cell viability in our case.

**Table 1:** Magnetic nanoparticle influence on cell viability.

Magnetic Nanoparticles		Cell Viability		Reference
Uncoated magnetite	0.01 to 0.40 mg/mL	HeLa cancer cells	Fluorescence microscopy	Li et al. (2012) [55]
Uncoated magnetite	0.01 to 0.5 mg/mL	Neural PC12 cells	XTT test	Marcus et al. (2016) [56]
Carboxymethyl dextran-magnetite	0.3–1.5 mg/mL	Caco-2 and MCF-7 human cancer cells.	Spectrofluorimetric assay	Rodríguez-Luccioni et al. (2011) [57]
DMSA coated magnetite	0.05–0.4 mg/mL	MCF-7 breast cancer cells	MTT test	Calero et al. (2015) [59]
Polymer coated magnetite	<200 µg/mL	V79 hamster lung fibroblasts	MTT test	Zavisova et al. (2015) [60]

And the study of MCF-7 breast cancer cells treated with magnetic nanoparticles (by MTT test) showed that concentrations ten thousand times lower than that used by us only induced slight reducing of cell viability, which is statistically non-significant [59]. As for the V79-4 cell line studied by us, in [6] it was demonstrated that concentrations of Fe_3_O_4_ ten times lower than in our experiments were able to reduce to half the cellular viability []. Magnetic nanoparticles can interact with the studied cells in two ways: (i) the nanoparticles attach to the cell membrane and block pores or ion channels disturbing cell metabolism and viability [61]; (ii) the nanoparticles enter the cells (endocytosis) where their catalytic effect leads to the generation of Reactive Oxygen Species (ROS) that underlie the development of oxidative stress. Free radicals are produced by the catalytic action of iron ions at the nanoparticle–cytoplasm interface [62]. Some authors have mentioned that magnetic nanoparticles could be nontoxic to living cells, mainly at low concentrations and depending on their surface modification with organic molecules [63], which is important for theranostic applications where protecting healthy cells is as important as destroying malignant ones.

On the one hand, coating the surface with certain biomolecules could improve biocompatibility, while on the other hand, releasing certain low levels of iron ions from the surface of magnetic nanoparticles could be beneficial for cellular metabolism [64].

We have assumed that the contribution of the degradation products to the decrease in cell viability is considerably lower than that of Rhodamine B since they have higher frontier orbital energy gap and thus are less reactive.

We found that degraded Rhodamine B solutions always had a weaker effect on cell viability compared to undegraded dye samples and we believe that we can consider that only these two compounds influenced cell growth and metabolism. However, we did not ignore the fact that although V79-4 cells are not known to be a source of secondary metabolites, their metabolic activity (as in the case of other cultured cells) could sometimes influence the pH of the culture medium during incubation, but only if the buffer system is counteracted, which may affect their cell growth and functions [65].

In our experiments, the carbon dioxide supply (5%) was continuously checked to maintain uniformly optimal conditions (neutral pH) for the cell culture throughout the incubation. However, if a pH deviation had occurred, it would have affected the viability of the control cell cultures in the same way as in the test samples.

The fact that, in both cases of degraded solutions, we have less pronounced cytotoxic effects in the cell culture samples compared to initial dye solutions is also consistent with the results of computational modeling on degradation products, which coexist in degraded solutions with intact Rhodamine B molecules, and whose chemical reactivity parameters indicate greater stability (and lower reactivity) than Rhodamine B.

**Figure 20:**
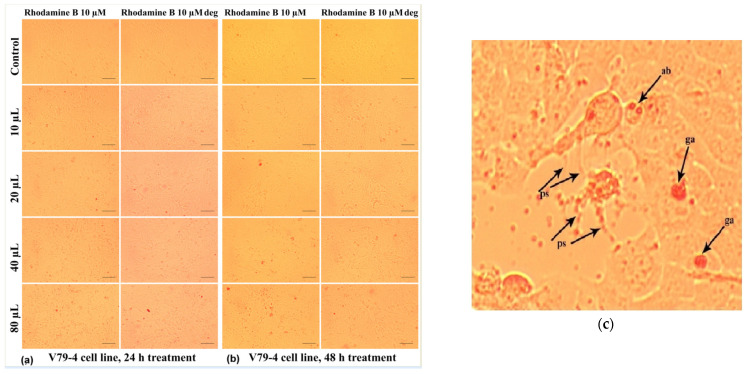
Morphological aspects of V79-4 fibroblasts treated with Rhodamine B 10 µM, evaluated by photon microscopy (10× objective, ruler 100 µm): (**a**) at 24 h and (**b**) at 48 h. (**c**) Detail image presenting the morphological changes evidenced during microscope investigation of V79-4 cells treated with Rhodamine B 10 µM. ps—filamentous pseudopod; ga—granular agglomerations; and ab apoptotic bodies.

Thus, their influence on V79-4 living cells is less harmful at any of the doses considered for this study. As can be seen in Figure 20a, the treatment with Rhodamine B 10 µM at 24 h generally induced a decrease in the number of cells adhered to the surface of the culture plate for doses increasing from 20 µL/mL to 80 µL/mL.

At the cellular level, other alterations of the normal morphology can also be identified (Figure 20c), such as the loss of the intercellular connection, with the pseudopods, (which ensure the junction between the cells) sometimes having the appearance of very thin filaments (“ps” in Figure 20c). In addition, Figure 20c shows the accumulation in the culture medium of some reddish inclusions, in the form of spheroid formations with a granular appearance (“ga”), which are in greater numbers for the relatively higher doses of 40 µL/mL and 80 µL/mL; those could consist in dye aliquots not metabolized by the cells.

In some cases (Figure 20c), cells whose cytoplasmic content had a granular appearance could be highlighted, which allows us to assume the existence of an apoptotic process through the formation of apoptotic bodies (“ab”) characterized by individualized vesicles and delimited by the cell membrane. At the same time, these cells were smaller in size, which indicates the phenomenon of pyknosis characterized by chromatin condensation (Figure 20c).

Thus, not the entire toxic potential of dye molecules is converted in lethal action.

In the case of the degraded Rhodamine B 10 µM, at 24 h (Figure 20a), the cellular changes identified under the microscope are of lower frequency in the selected photos, but in the culture medium, the presence of a greater number of colored granules is observed. Extending the treatment up to 48 h had the effect of intensifying the cytotoxic effects induced by Rhodamine B 10 µM, the phenomena also being visible in the case of degraded Rhodamine B 10 µM (Figure 20b).

This response may be due to the inability of cells to neutralize the action of this dye through specific physiological mechanisms. However, at 48 h, in the case of Rhodamine B 10 µM compared to degraded Rhodamine B 10 µM, the reduction in colored inclusions, both individual ones and agglomerated forms, could be noticed.

**Figure 21:**
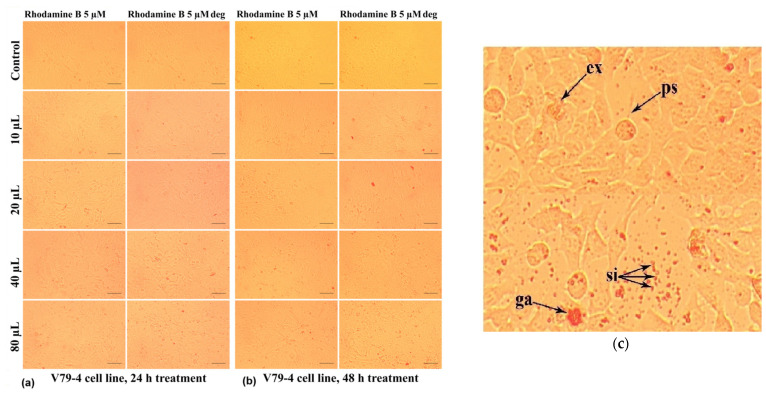
Morphological aspects of V79-4 fibroblasts treated with Rhodamine B 5µM, evaluated by photon microscopy (10× objective, 100 µm ruler). (Rhodamine B deg. = Rhodamine B degraded): (**a**) at 24 h and (**b**) at 48 h. (**c**) Detail on the V79-4 fibroblast morphology after treatment with Rhodamine B 5 µM, 80 µL/mL for 48 h. ga—granular agglomerations; ps—filamentous pseudopods covered by small red inclusion; si—singular inclusions; and ex—expelled cellular material.

The response of the cells to the treatment with Rhodamine B 5 µM and degraded Rhodamine B 5 µM, both after 24 h (Figure 21a) and after 48 h of treatment (Figure 21b), was evidenced by cytomorphological changes in smaller frequency, determined by the use of a lower concentration of Rhodamine B. There were some reddish inclusions, in the form of both small, individualized particles (“si”) and granular agglomerations (“ga”), and filamentous pseudopods (“ps“) and expelled cellular material ("ex") (Figure 21c).

The results highlighted by the microscopic analyses are consistent with those obtained in the case of the MTT test, the degree of cytotoxicity exerted by Rhodamine B being increased with the applied dose and with the extension of the treatment duration.

There are some examples of studies of this kind in the specialized literature (Table 2). A series of tests performed on cultures of human fibroblasts isolated from the lips (KD cells) demonstrated the cytotoxic effect of Rhodamine B applied in doses of 25 and 50 µg/mL, manifested by the decrease in cell proliferation, the detachment of cells from the substrate, the inhibition of synthesis of collagen and glycosaminoglycan [34,66], which induced disruption of lip wound healing and delayed repair of damaged tissue. The study was carried out in the context of the use of this dye in cosmetics, through its inclusion in products applicable to the lips. In the same context, Rhodamine B was shown to induce ovarian toxicity, the diminution of the follicles number, and the lowered thickness of endometrium [25].

Recent studies show induction of oxidative stress and enhancement of uterine epithelial cell proliferation as a result of the effects of Rhodamine B injected into Wistar mice [67]. On the other hand, the studies carried out by supplying Rhodamine B in the food of the wild mouse species *Rattus norvegicus* [68] highlighted the change in the expression of the genes responsible for controlling the apoptotic process in the cerebellum and brainstem tissue.

Through this research, attention is drawn to the risk of using this dye in cosmetics or the food industry, being banned in California, as a result of the possible carcinogenic effect. Discussing the toxicity of degradation products of Rhodamine B, special attention can be paid to benzoic acid, a natural phenolic compound, which is also synthesized for various utilizations in cosmetics and the food industry. Its antimicrobial effect on *Escherichia coli* bacterial cells was demonstrated in [69]. The ability of benzoic acid to decrease viability in various cancerous cell cultures in comparison to normal kidney epithelial cells is also highlighted by the MTT test [70]; it is expected to be able to induce some bioeffects in the healthy fibroblast cells chosen for our study.

**Table 2:** Cytotoxic effects of Rhodamine B.

Biological Material	Rhodamine B Effect	Reference
Human fibroblasts isolated from the lips (KD cells)	Decreased cell proliferation, inhibition of collagen synthesis	Kaji et al. (1991) [34] Kaji et al. (1992) [66]
Cerebellum and brainstem tissue of *Rattus norvegicus*	Cell apoptosis	Sulistina et al. (2020) [68]
*H. verticillata* cells	Inhibitory effects on photosystem II	Sharma et al. (2022) [26]
Uterine cervix of Wistar albino rats	Lipid peroxidation	Safitri et al. (2015) [67]
Zebrafish	Reproductive toxicity	Priya et al. (2024) [23]
Wistar rats	Ovarian toxicity, decreased follicles number	Maryanti et al. (2014) [25]

Phthalic acid shows significant toxicity (according to in vitro and in vivo studies) at the level of reproductive activity, can be a mutagenesis factor, and can even be taken as a toxicity biomarker [71] for those working in environments where they are exposed to phthalic acid and its esters (cosmetics, perfumes, food packaging, toys, and even some medical utensil industries). For example, repeated administration of phthalic acid in the feed of mice and rabbits has been shown to have undesirable effects on blood level.

On the other hand, it was found that benzyloxyamine has an indirect effect of compensating the action of free radicals, that is, it is a scavenger of some degradation products of oxidative stress [72]. In addition, no antibacterial activity could be detected for benzyloxyamine [73].

Rhodamine B interacts with living cells through membrane transport and then accumulates in organelles such as mitochondria [74] or lysosomes, inducing oxidative stress and damaging DNA and proteins. This results in lipid peroxidation, cell cycle arrest [67], and cell apoptosis [68].

Its degradation products seem to be less toxic. Benzoic acid has antibacterial and antifungal activity (in *E. coli* and yeasts) as well as anticancer effects [70], and has been widely used in the feed industry, at relatively low concentrations, as an organic acidifier and conserving ingredient but avoiding high concentrations that can lead to metabolic or physiological disturbances [75]. Some authors studied the toxicity of phthalic acid esters that were found to harm the nervous and reproductive systems of mammals [76].

Thus, the contribution of this compound to the global cytotoxicity of Rhodamine-B-degraded solutions seems to be insignificant, or rather, it could even compensate for part of the solution’s global cytotoxicity.

## 3. Materials and Methods

### 3.1. Methods of Magnetic Nanoparticles Investigation

We applied an adapted synthesis method, after [77], the magnetite (Fe_3_O_4_) nanopowder being prepared by chemical co-precipitation from ferric chloride (3.62 g in 100 mL of distilled water) and ferrous sulfate (1.86 g in 100 mL of distilled water). We performed the synthesis of nanoparticles by slowly adding the alkaline agent (hot 1.7 M sodium hydroxide) to the reagent mixture, at a stoichiometric ratio and at a reaction temperature of approximately 80 °C to avoid the formation of agglomerates by the particles. The ferrophase was decanted and washed three times with a total volume of 300 mL of hot distilled water to remove impurities or traces of unreacted salts.

The nanoparticles were carefully dried in a partial vacuum oven at approximately 75 °C and used as such, without a protective coating.

A UHR-TEM LIBRA®200MC (Carl Zeiss GmbH, Oberkochen, Germany) device for Scanning Transmission Electron Microscopy was used to perform microstructural investigation and the image analysis was performed using ImageJ software (vers.1.8.0) for nanoparticle size measurement. The nanoparticle crystallinity in the 20–80° range was analyzed with a Shimadzu Lab XRD-6000 device (Shimadzu Corporation, Kyoto, Japan) working in Bragg–Bentano geometry and using Cu–Kα radiation with a wavelength of 1.5406 Å. The 2θ values were recorded with a scanning step of 0.02° and a 0.5 °/min scan speed. FTIR spectra recording was carried out with an FTIR VERTEX 70 (Bruker Optics Company, Bremen, Germany), with a 0.5 cm^−1^ resolution on KBr pellets. The powder of magnetic nanoparticles for the dye treatment was weighted with semi-analytical balance (10^−4^ g accuracy) and the dye solution aliquots were manipulated with automated micropipettes, the volume of the reaction sample being adjusted at 10 mL.

### 3.2. Rhodamine B Samples

#### 3.2.1. Rhodamine B Wastewater Models

Two models of wastewater, loaded with Rhodamine B, were used to study the cytotoxicity on some mammalian cells, i.e., Rhodamine B 10 µM and Rhodamine B 5 µM. 10 mL volume from each solution was used in each experiment. Degradation rate was calculated as

(5) $$D(\%)=100\frac{\left(A_{0}-A\right)}{A_{0}}$$

where *A* is the initial absorbance of the treated solution and *A*_0_ is its initial absorbance.

#### 3.2.2. Rhodamine B Solution Irradiation

The source of ultraviolet radiation was a commercially available disinfection tube with 30 W power, emitting 12 W in the UV-C range (254 nm), with the 10 mL samples being poured in an open 3 cm diameter dish at 25 cm under the irradiation tube.

### 3.3. Theoretical Reactivity Modeling

The quantum chemical study of Rhodamine B and its degraded products was carried out with the PM3 method (Parametric Method 3), a semi-empirical method for the quantum calculation of the molecular electronic structure in computational chemistry, through computational algorithms implemented in the program package Spartan 18, used by us [78].

### 3.4. Biological Material

The biological material used in the in vitro experiments, to evaluate the effects of the mentioned compounds on cellular processes, has consisted in normal cell cultures, namely lung fibroblasts, line V79-4 (ATCC CCL-93) of the Chinese hamster *Cricetulus griseus* (with relatively high response efficiency and short regeneration time (12–14 h)). The cells were cultured in DMEM medium (Dulbecco’s Modified Growth Medium, PAN-BiotechGmbH, Aidenbach, Germany) supplemented with 10% fetal bovine serum (EurocloneS.p.A., Milan, Italy) and antibiotic solution (penicillin 100 μg/mL and streptomycin 100 IU/mL—Capricorn ScientificGmbH, Ebsdorfergrund, Germany), grown in an incubator (Binder GmbH, Tuttlingen, Germany) at 37 °C, in a humid atmosphere with 5% CO_2_ [79].

V79 is a widely used cell line for studying the effects of external compounds and often involves exposing V79-4 cells to various chemicals to assess their cytotoxicity, mutagenicity, and metabolic activation, using the cells as a model to understand how these compounds affect living systems.

They are a widely used and established cell line in biological research because they have a shortened cell cycle and are particularly useful for genetic toxicity tests due to their stable karyotype and morphology. V79 cells have been widely used in studies of the effects of various chemicals and compounds on cell growth and viability. Their extensive use in research attests to their utility and importance in biology [80].

### 3.5. Experimental

#### 3.5.1. Rhodamine B Photodegradation

The changes undergone by the two Rhodamine B solutions were spectrally analyzed using a Shimadzu device type 177 Pharma Spec (Shimadzu Corporation, Kyoto, Japan), after the photodegradation treatment with magnetite powder, 4 and 8 g/L, in the presence of hydrogen peroxide (10 and 20 mM) and UV-C radiation (30–60–120 min exposure time).

#### 3.5.2. Cell Culture and Treatment

The cytotoxicity test was based on solutions of Rhodamine B and Rhodamine B degraded with magnetite. We worked by having kept a permanent magnet at the vial bottom before we have extracted Rhodamine B degraded solution samples for cytotoxicity assay.

The assessment of the impact on cell viability was carried out by the colorimetric method with 3-(4,5-dimethylthiazol-2-yl)-2,5-diphenyl tetrazolium bromide (MTT), adapted from [81,82,83], a method based on the ability of mitochondrial dehydrogenases in living cells to convert the yellow water-soluble substrate (MTT) into dark blue, water-insoluble formazan. The resulting amount of formazan is directly proportional to the number of living cells [84]. V79-4 cells were detached with trypsin/EDTA, then counted and resuspended in 96-well microplates (6.5 × 10^3^ cells/well) and maintained at the same temperature and humidity conditions.

After the formation of the monolayer (24 h), the cells were exposed for 24 and 48 h to the studied Rhodamine B variants, the solutions being added to the culture medium in the following volumes: 10, 20, 40, 80 µL/mL.

After completion of treatment, cells were processed according to the MTT assay and absorbance was measured at 570 nm using the Biochrom EZ Read 400 automated microplate reader (Biochrom Ltd., Cambridge, UK).

Cell viability was calculated according to the following formula:

(6) $$Cell\;viability\left(\%\right)=E_{sample}/E_{control}\times 100$$

where E_sample_ is the absorbance (light extinction) of the sample and E_control_ is the extinction of the untreated control.

#### 3.5.3. Microscopic Study

Microscope investigations were accomplished with inverted microscope Nikon Eclipse TS 100 (Nikon Corporation, Tokyo, Japan), equipped with digital camera MshOt MS60, to identify the modifications induced in the culture medium and especially in the cell morphology.

### 3.6. Statistical Analysis

All variants were analyzed in triplicate, and results are presented as average ± standard deviation (StDev). The significance of the difference between the average values was analyzed using ANOVA statistical test [85].

## 4. Conclusions

A combined treatment of wastewater models loaded with Rhodamine B was applied using magnetic nanoparticles, UV radiation, and hydrogen peroxide, obtaining promising results in terms of initial dye depletion. The quantum chemical modeling of the studied molecule evidenced moderate photodegradability and chemical reactivity based on the 6 eV energy gap of the frontier orbitals. Cytotoxicity was assessed with MTT viability test performed on V79-4 cell line and 20 to 80 µL/mL solution doses that revealed approx. 60% survival for 10 µM degraded Rhodamine B and about 74% survival for 5 µM degraded Rhodamine B at 80 µL/mL dose. The study of cell morphology in the cultures treated with dye solutions revealed various changes that, in general, were more abundant for Rhodamine B 10 µM compared to Rhodamine B 5 µM and the corresponding degraded variants, foreshadowing cell impairing which leads to apoptosis. To extend the proposed method at the large scale, the monitoring of reagent intimate interaction should be ensured with adequate tools able to provide conditions of uniformity and homogeneity within the designed reactor.

## Data Availability

All of the data is contained within the article.
